# Fine Mapping of QTLs for Ascochyta Blight Resistance in Pea Using Heterogeneous Inbred Families

**DOI:** 10.3389/fpls.2017.00765

**Published:** 2017-05-09

**Authors:** Ambuj B. Jha, Krishna K. Gali, Bunyamin Tar’an, Thomas D. Warkentin

**Affiliations:** Crop Development Centre – Department of Plant Sciences, University of Saskatchewan, SaskatoonSK, Canada

**Keywords:** ascochyta blight, genotyping-by-sequencing, heterogeneous inbred family, quantitative trait loci, *Pisum fulvum*, *P. sativum*

## Abstract

Ascochyta blight (AB) is an important disease of pea which can cause severe grain yield loss under wet conditions. In our previous study, we identified two quantitative trait loci (QTLs) abIII-1 and abI-IV-2 for AB resistance and these QTLs were consistent across locations and/or years in an inter-specific pea population (PR-19) developed from a cross between Alfetta (*Pisum sativum*) and P651 (*P*. *fulvum*). The objectives of this study were to fine map the abIII-1 and abI-IV-2 QTLs using a high density single nucleotide polymorphism (SNP)-based genetic linkage map and analyze identified markers in heterogeneous inbred family (HIF) populations. Selective genotyping of 51 PR-19 recombinant inbred lines was performed using genotyping-by-sequencing (GBS) and the resulting high density genetic linkage map was used to identify eight new SNP markers within the abI-IV-2 QTL, whereas no additional SNPs were identified within the abIII-1 QTL. Two HIF populations HIF-224 (143 lines) and HIF-173 (126 lines) were developed from F_6_ RILs PR-19-224 and PR-19-173, respectively. The HIF populations evaluated under field conditions in 2015 and 2016 showed a wide range of variation for reaction to AB resistance. Lodging score had significant positive (*P* < 0.001) correlation with AB scores. HIFs were genotyped using SNP markers within targeted QTLs. The genotypic and phenotypic data of the HIFs were used to identify two new QTLs, abI-IV-2.1 and abI-IV-2.2 for AB resistance within the abI-IV-2 QTL. These QTLs individually explained 5.5 to 14% of the total phenotypic variation. Resistance to lodging was also associated with these two QTLs. Identified SNP markers will be useful in marker assisted selection for development of pea cultivars with improved AB resistance.

## Introduction

Ascochyta blight (AB), caused by *Peyronellaea pinodes* (Berk. & A. Bloxam) Aveskamp, Gruyter & Verkley (Aveskamp et al., 2010), is the most important pea (*Pisum sativum*) disease which can severely affect grain yield under wet conditions in most pea growing regions in the world (Lawyer, 1984; Xue et al., 1997; Kraft et al., 1998). The impact of the disease under field conditions is greatly affected by agronomic traits including lodging and plant height (Tar’an et al., 2003; Banniza et al., 2005; Le May et al., 2009; Jha et al., 2013, 2016). Genetic resistance is the optimal approach to reduce the disease impact (Zimmer and Sabourin, 1986). More than 3500 cultivated pea accessions were evaluated for their reaction to the disease resulting in the identification of a few lines with low to moderate levels of resistance (Kraft et al., 1998; Zhang et al., 2006). In contrast, a higher level of resistance was identified in wild pea (*P*. *fulvum*) accessions (Clulow et al., 1991; Wroth, 1998; Fondevilla et al., 2005; Jha et al., 2012). Further, Fondevilla et al. (2005) reported the highest level of resistance in accession P651 (*P. fulvum*) compared to other wild peas, P670 (*P. sativum* ssp. *elatius*) and P665 (*P. sativum* ssp. *syriacum*). Promising accessions (*P. fulvum* and *P. sativum* ssp. *elatius*) were identified upon evaluation of 44 wild pea accessions which had the potential for improvement of AB resistance (Jha et al., 2012). Among them, the most promising accession, P651 (*P. fulvum*) was utilized for resistance breeding (Sindhu et al., 2014; Jha et al., 2016).

Previously, more than 30 quantitative trait loci (QTLs) were identified for resistance to AB in *P*. *sativum* mapping populations on all seven linkage groups (LGs) under field or controlled conditions (Timmerman-Vaughan et al., 2002, 2004; Tar’an et al., 2003; Prioul et al., 2004). QTLs were also identified in a cross involving wild pea, *P. sativum* subsp. *syriacum* (Fondevilla et al., 2008, 2011; Carrillo et al., 2014). Co-localization of QTLs for disease resistance with candidate genes including *RGAs* (resistance gene analogs), *PsDof1* (a putative transcription factor) and *DRR230-b* (a pea defensin) involved in defense responses to *P*. *pinodes* was reported in pea (Timmerman-Vaughan et al., 2002, 2016; Prioul-Gervais et al., 2007). Further, Jha et al. (2015) reported significant association of SNPs detected within candidate genes *PsDof1* (PsDof1p308) and *RGA-G3A* (RGA-G3Ap103) with AB scores. Most recently, nine QTLs were identified for AB resistance in an inter-specific pea population (PR-19) developed from a cross between Alfetta (*P*. *sativum*) and wild pea accession P651 (*P*. *fulvum*) (Jha et al., 2016). These QTLs individually explained 7.5 to 28% of the phenotypic variation.

Quantitative trait loci mapping studies in several pea crosses have resulted in the identification of genomic regions associated with AB resistance, however, these QTLs cover large regions which may not be effective for marker-assisted selection (MAS). Though several markers linked to resistance genes have been identified, even the closest markers are not necessarily tightly linked to the gene of interest (reviewed by Michelmore, 1995). Recombination could occur between a marker and QTL if markers are not tightly linked to genes (Collard et al., 2005). High-resolution or fine mapping of QTLs can be used to identify more tightly-linked or perfect markers within the gene sequence that can be efficiently utilized for MAS (reviewed by Mohan et al., 1997). Development of an advanced population, such as near isogenic lines (NILs), is required for fine mapping. Conventional consecutive backcrossing method was the original method for NIL development. Tuinstra et al. (1997) proposed development of heterogeneous inbred family (HIF) populations, an alternative, more efficient method than the NILs. This approach has been widely used in several species including Arabidopsis, soybean and maize for fine mapping of QTLs (Meng et al., 2008; Bai et al., 2010; Todesco et al., 2010; Coles et al., 2011; Dwiyanti et al., 2011; Watanabe et al., 2011; Bouteillé et al., 2012).

Among the nine AB resistance QTLs identified in PR-19 population, two QTLs abIII-1 and abI-IV-2 were consistent across locations and/or years (Jha et al., 2016). The objectives of this research were to identify additional SNP markers within abIII-I and abI-IV-2 QTLs and to fine map them using HIF populations for identification of closely linked markers for AB resistance in pea.

## Materials and Methods

### Plant Material

Previously, PR-19 recombinant inbred line (RIL) population was generated from a cross between Alfetta (*P*. *sativum*) and P651 (*P*. *fulvum*) (Sindhu et al., 2014). P651 (original code IFPI3232) was first identified in Syria, then characterized by Consejo Superior de Investigaciones Científicas (Cordoba, Spain). For fine mapping of QTLs abI-IV-2 and abIII-1, HIF populations HIF-224 and HIF-173 were developed from F_6_ RILs of PR-19-224 and PR-19-173, respectively.

### Selection of PR-19 Lines for HIF Populations

RILs PR-19-57, PR-19-132, PR-19-176, and PR-19-224 segregated for marker loci associated with the QTL abI-IV-2, and PR-19-04, PR-19-65, PR-19-115, and PR-19-173 segregated for marker loci associated with the QTL abIII-1. Three seeds of each of these RILs were sown in 2 gallon pots in a greenhouse with 22 + 3°C day/20 + 3°C night temperature under an 18-h photoperiod with approximately 60% relative humidity. Genomic DNA was extracted from freeze-dried leaf tissue collected from each plant using DNeasy Plant Mini Kit (QIAGEN Inc., Valencia, CA, USA) and used for Kompetitive Alelle Specific PCR (KASP) assays to validate heterozygous alleles for SNP loci within the QTLs. Allele-specific primers were designed for SNP loci PsC8780p118 (abIII-1) and PsC6805p316 (abI-IV-2) (Supplementary Table S1) using Primer-Picker software (LGC Genomics, Beverly, MA, USA). A total reaction volume of 10 μl was prepared by adding 20 ng of template DNA, 5 μl of KASP 2X Reaction Mix and 0.14 μl of KASP assay mixture (LGC Genomics, Beverly, MA, USA) in a 96-well plate format. Amplifications were performed using StepOnePlus Real-Time PCR system (Applied Biosystems, USA) according to the program described in Jha et al. (2015). Genotypic data were analyzed using SNPViewer software (LGC Genomics, Beverly, MA, USA).

### Development of HIF-224 and HIF-173

**FIGURE 1:**
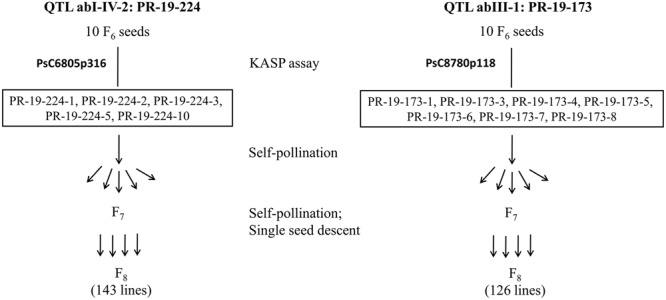
**Development of heterogeneous inbred family (HIF) populations, HIF-224 and HIF-173 from lines PR-19-224 and PR-19-173, respectively**.

Ten F_6_ seeds each for PR-19-224 and PR-19-173 were grown under greenhouse conditions and tested for heterogeneity by KASP assays as described earlier. Based on these assays, five seeds for PR-19-224 and seven seeds for PR-19-173 had heterozygous alleles for markers associated with abI-IV-2 and abIII-1, respectively. Seeds were bulked from five plants of PR-19-224 and seven plants of PR-19-173. Using single seed descent, self-pollination and bulking of seeds were conducted for F_7_ to F_8_ generation. Progenies at F_8_ were represented HIF-224 and HIF-173 for PR-19-224 and PR-19-173, respectively (**Figure 1**).

### Assessment of AB Resistance and Other Agronomic Traits Under Field Conditions

HIF-224 (143 lines) and HIF-173 (126 lines) along with parental checks (Alfetta and P651) were evaluated for reaction to AB and other agronomic traits including days to flower (DTF), plant height, lodging, days to maturity (DTM), and grain yield on a plot basis under field conditions in 2015 at Saskatoon with two replicates, and in 2016 at Saskatoon and Rosthern with three replicates at each location. The experimental design was a randomized complete block design with three-row plots of 1.0 m × 1.0 m, a plant density of 75 plants m^-2^ and row spacing of 0.25 m. Plants were inoculated at the start of the flowering stage with approximately 3 g per plot of pea straw that had been naturally infected by *P. pinodes* in the previous season, air dried, and chopped into approximately 2-cm pieces. HIFs were evaluated for AB severity at pod filling and physiological maturity stages (80% of pods in the plot turned brown) using a scale of 0 (no disease) to 9 (whole plant severely blighted) based on Xue et al. (1996). Lodging was assessed on a 1 (upright) to 9 (completely lodged) scale. Plant height was measured from the soil level to the tip of the central stem at physiological maturity. DTF and DTM were calculated as the number of days from planting to 50% bloom and physiological maturity, respectively.

### Identification of Additional SNPs in QTLs

A high density genetic linkage map of PR-19 based on selective genotyping of the RIL population was developed for identification of additional SNP loci within the two targeted QTLs. Fifty-one F_7_ RILs of PR-19 including PR-19-224 and PR-19-173 along with the parents (Alfetta and P651) were genotyped using genotyping-by-sequencing (GBS) method as described by Elshire et al. (2011). Twenty ng/μL DNA of each RIL as quantified using picogreen was digested with restriction enzymes *Pst*I and *Msp*I. Digested DNA of individual RILs was ligated with a unique 4 to 8 base pair barcode adapter. At this stage the DNA samples were pooled for construction of a single library for sequencing. Paired-end sequencing of the library was done in a single lane of an Illumina Hiseq sequencer using V4 sequencing chemistry.

The raw sequence reads were assigned to individual RILs based on the ligated barcode adapter. Following this deconvolution, barcode sequences were removed from the sequence. The reads were then trimmed for quality with Trimmomatic-0.33, and mapped to the draft genome assembly provided through the pea genome sequencing consortium (Madoui et al., 2016) using Bowtie2-2.2.5. SNP variants were identified and converted to VCF format using Samtools-1.1 and BCFtools-1.1.

After filtering for missing values and heterozygosity, 6160 SNP markers were selected for linkage analysis. Segregation data of these markers were combined with 733 polymorphic SNP markers previously genotyped using Illumina GoldenGate 1536 SNP array (Jha et al., 2016). Combined SNP marker segregation data were used for linkage analysis using MstMap. SNP markers from the GoldenGate assay served as anchor markers to identify additional SNP loci within the targeted QTLs. All the SNP markers identified within QTLs were converted to KASP assays (Supplementary Table S1) and used for genotyping of the complete set of 144 RILs of PR-19 for cross-validation of their genetic linkage positions.

### Genotyping of HIF-224 and HIF-173

Genomic DNA was extracted from freeze dried leaf tissues collected from single plants of HIF-224 (143 lines) and HIF-173 (126 lines) progenies using DNeasy Plant Mini Kit (QIAGEN Inc., Valencia, California, USA). HIF-224 lines were genotyped using 20 SNP markers (Supplementary Table S1) representing the QTL abI-IV-2 and the region adjacent to the QTL by KASP assays. HIF-173 population segregating for QTL abIII-1 was genotyped with three SNP markers, PsC22609p103, PsC8780p118, and PsC23317p284, each representing a unique locus within this QTL.

### Linkage Mapping and QTL Analysis in HIF Populations

The linkage map was constructed separately for PR-19, HIF-224, and HIF-173 using MAPMAKER (Lander et al., 1987). QTL mapping was performed by composite interval mapping (CIM) using Windows QTL Cartographer 2.5 (Wang et al., 2012). The significance threshold (*P* < 0.05) was used to declare the presence of QTLs by performing 1000 permutations of the data (Churchill and Doerge, 1994). MapChart 2.2 was used for graphical presentation of linkage maps (Voorrips, 2002).

### Statistical Analysis

PROC MIXED implemented in SAS^®^9.3 (SAS Institute Inc. Cary, NC, USA) was used for data analysis. Line was treated as a fixed effect whereas replication was treated as a random effect across the HIFs. Homogeneity of variance test (HOVTEST) was used to assess the homogeneity of variance among replications.

## Results

### Selection of PR-19 Lines for HIF Populations

Four RILs each tested for abI-IV-2 (PR-19-57, PR-19-132, PR-19-176, and PR-19-224) and abIII-1 (PR-19-04, PR-19-65, PR-19-115, and PR-19-173) had heterozygous alleles within QTLs, i.e., these lines were segregating for markers associated with AB, which is a prerequisite for HIF development. On the basis of KASP assays and AB scores of lines, PR-19-224 and PR-19-173 were selected for development of HIF-224 and HIF-173, respectively.

### Assessment of AB Resistance and Other Agronomic Traits under Field Conditions

**FIGURE 2:**
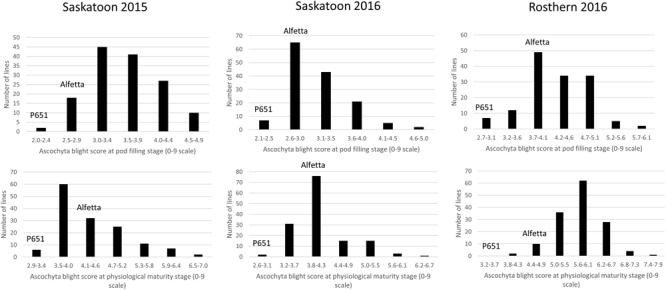
**Frequency distribution of 143 lines of HIF population, HIF-224 using least square means of Saskatoon 2015, Saskatoon 2016 and Rosthern 2016 for the reaction to ascochyta blight (AB) resistance at pod filling and physiological maturity stages under field conditions**.

**FIGURE 3:**
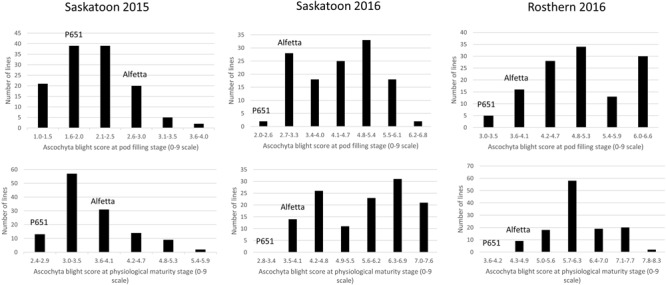
**Frequency distribution of 126 lines of HIF population, HIF-173 using least square means of Saskatoon 2015, Saskatoon 2016 and Rosthern 2016 for the reaction to AB resistance at pod filling and physiological maturity stages under field conditions**.

**FIGURE 4:**
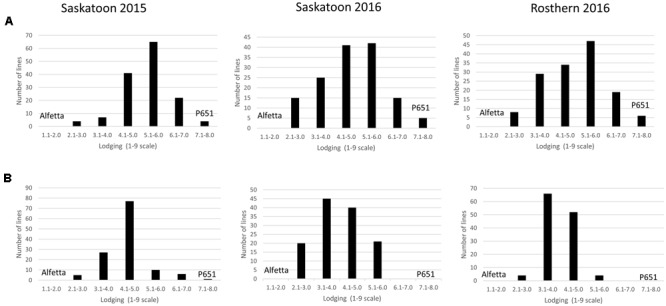
**Frequency distribution of 143 lines of HIF population, HIF-224 (A)** and 126 lines of HIF-173 **(B)** using least square means of Saskatoon 2015, Saskatoon 2016 and Rosthern 2016 for lodging at physiological maturity stage under field conditions.

HIF-224 and HIF-173 showed a wide range of variation for reaction to AB, plant height, lodging, and grain yield under field conditions in 2015 at Saskatoon and in 2015 and 2016 at Saskatoon and Rosthern locations in Saskatchewan (**Tables 1, 2** and **Figures 2**–**4**). Data from different station years could not be combined for analysis of variance due to significant effect of locations and years in the HOVTEST. In general, the effect of line was significant (*P* < 0.05) for AB scores, plant height, lodging, and grain yield. AB scores of HIF-224 ranged from 2 to 7 at pod filling, and 2 to 8 at physiological maturity (0–9 scale), whereas for HIF-173, scores ranged from 1 to 7 at pod filling, and 2 to 9 at physiological maturity. Alfetta had disease score of 3 to 4 at pod filling and 4 to 5 at physiological stage, whereas P651 had disease score of 2 to 3 and 3 to 4 at pod filling and physiological maturity stage, respectively. Lodging scores varied from 1 to 9 for HIF-224, whereas for HIF-173, scores varied from 1 to 7 on the 1–9 scale. Alfetta had 1 to 3 lodging score whereas P651 had 8 to 9 score. HIFs had a small range of variation for DTF and DTM at different station years, while plant height and grain yield had a wide range of variation among tested HIF lines. For both HIFs, AB scores were positively correlated with lodging (*P* < 0.001) and negatively correlated with plant height (*P* < 0.001) and grain yield (*P* < 0.01) (**Tables 3, 4**).

**Table 1:** *F*-values, coefficients of variations (CV) of statistical analyses, and means with standard deviations (SD) of ascochyta blight (AB) scores and other agronomic assessments for 143 lines of heterogeneous inbred family (HIF) population, HIF-224 evaluated under field conditions in 2015 at Saskatoon and in 2016 at Saskatoon and Rosthern, Saskatchewan.

		AB1 (0–9 scale)	AB2 (0–9 scale)	Days to flower (DTF)	Plant height (cm)	Lodging (1–9 scale)	Days to maturity	Grain yield (Kg/ha)
Saskatoon	Line	1.4^∗^	1.9^∗∗∗^	0.7NS	2.6^∗∗∗^	1.8^∗∗∗^	1.0NS	0.9NS
2015	Range	2–6	2–8	36–39	29–54	2–8	75–79	45–1154
	Mean ± SD	3.4 ± 0.8	4.4 ± 1.1	37.4 ± 0.7	44.5 ± 5.0	5.6 ± 0.9	77.6 ± 1.4	416 ± 218
	CV (%)	23.8	25.0	2.0	11.2	15.8	1.8	52.4

Saskatoon	Line	1.9^∗∗∗^	5.1^∗∗∗^	0.9NS	1.9^∗∗∗^	3.5^∗∗∗^	1.3NS	2.7^∗∗∗^
2016	Range	2–6	2–8	38–41	28–57	1–9	71–74	37–2912
	Mean ± SD	3.2 ± 0.7	4.5 ± 1.0	39.6 ± 0.9	44.2 ± 8.5	4.5 ± 1.8	72.6 ± 1.1	864 ± 66
	CV (%)	22.6	22.2	2.4	19.2	39.1	1.4	52.1

Rosthern	Line	2.3^∗∗∗^	2.0^∗∗∗^	1.1NS	2.9^∗∗∗^	5.1^∗∗∗^	1.2NS	3.2^∗∗∗^
2016	Range	2–7	3–8	40–44	28–60	1–9	80–84	58–2487
	Mean ± SD	4.2 ± 0.8	5.7 ± 0.9	41.7 ± 1.2	43.1 ± 6.4	5.1 ± 1.4	82.1 ± 1.2	1051 ± 59
	CV (%)	20.0	15.8	2.6	14.8	27.4	1.4	38.7

*NS- not significant; ^∗^*P* < 0.05; ^∗∗^*P* < 0.01; ^∗∗∗^*P* < 0.001; AB1 and AB2 denote AB scores at pod filling and physiological maturity stages, respectively.*

**Table 2:** *F*-values, coefficients of variations (CV) of statistical analyses, and means with standard deviations (SD) of AB scores and other agronomic assessments for 126 lines of heterogeneous inbred family (HIF) population, HIF-173 evaluated under field conditions in 2015 at Saskatoon and in 2016 at Saskatoon and Rosthern, Saskatchewan.

		AB1 (0–9 scale)	AB2 (0–9 scale)	DTF	Plant height (cm)	Lodging (1–9 scale)	Days to maturity	Grain yield (Kg/ha)
Saskatoon	Line	1.4^∗^	1.5^∗^	1.0NS	5.9^∗∗∗^	1.3NS	1.3NS	6.5^∗∗∗^
2015	Range	1–6	2–7	37–41	28–64	2–7	85–92	205–2886
	Mean ± SD	3.3 ± 0.7	4.1 ± 1.0	38.4 ± 1.0	54.0 ± 6.6	4.6 ± 0.7	89.3 ± 2.6	980 ± 507
	CV (%)	32.6	26.5	2.5	12.2	14.3	2.9	51.7

Saskatoon	Line	6.4^∗∗∗^	8.6^∗∗∗^	1.5^∗^	14.7^∗∗∗^	3.7^∗∗∗^	1.1NS	5.41^∗∗∗^
2016	Range	2–7	3–8	35–39	29–76	2–6	83–88	95–4126
	Mean ± SD	4.5 ± 1.2	5.7 ± 1.3	37.0 ± 1.2	62.4 ± 11.3	3.7 ± 1.1	85.1 ± 1.4	2008 ± 113
	CV (%)	26.4	22.3	3.1	18.2	27.5	1.6	38.4

Rosthern	Line	4.6^∗∗∗^	7.6^∗∗∗^	1.0NS	11.3^∗∗∗^	1.5^∗∗^	1.4^∗^	3.3^∗∗∗^
2016	Range	3–7	4–9	37–42	27–74	1–6	90–95	40–4858
	Mean ± SD	4.9 ± 1.1	6.6 ± 1.3	39.9 ± 1.2	55.3 ± 13.4	3.2 ± 0.8	92.6 ± 1.3	1602 ± 104
	CV (%)	20.4	19.2	2.7	24.3	25.1	1.4	44.3

*NS- not significant; ^∗^*P* < 0.05; ^∗∗^*P* < 0.01; ^∗∗∗^*P* < 0.001; AB1 and AB2 denote AB scores at pod filling and physiological maturity stages, respectively.*

**Table 3:** Pearson correlation coefficients for traits of 143 lines of heterogeneous inbred family (HIF) population, HIF-224 evaluated under field conditions in 2015 at Saskatoon and in 2016 at Saskatoon and Rosthern, Saskatchewan.

HIF-224	DTF	Plant height	Lodging	Days to maturity	Grain yield	AB1
Plant height	–0.01NS					
Lodging	–0.11NS	–0.52^∗∗∗^				
Days to maturity	0.92^∗∗∗^	0.01NS	–0.11NS			
Grain yield	0.04NS	0.69^∗∗∗^	–0.62^∗∗∗^	0.04NS		
AB1	–0.23^∗∗^	–0.53^∗∗∗^	0.67^∗∗∗^	–0.23^∗∗^	–0.64^∗∗∗^	
AB2	–0.24^∗∗^	–0.40^∗∗∗^	0.59^∗∗∗^	–0.23^∗∗^	–0.49^∗∗∗^	0.86^∗∗∗^

*NS-not significant; ^∗∗^*P* < 0.01; ^∗∗∗^*P* < 0.001; AB1 and AB2 denote AB scores at pod filling and physiological maturity stages, respectively.*

**Table 4:** Pearson correlation coefficients for traits of 126 lines of heterogeneous inbred family (HIF) population, HIF-173 evaluated under field conditions in 2015 at Saskatoon and in 2016 at Saskatoon and Rosthern, Saskatchewan.

HIF-173	DTF	Plant height	Lodging	Days to maturity	Grain yield	AB1
Plant height	–0.25^∗∗^					
Lodging	0.18^∗^	–0.48^∗∗∗^				
Days to maturity	0.77^∗∗∗^	–0.12NS	0.16NS			
Grain yield	–0.09NS	0.56^∗∗∗^	–0.44^∗∗∗^	–0.12NS		
AB1	0.28^∗∗^	–0.65^∗∗∗^	0.46^∗∗∗^	0.23^∗^	–0.28^∗∗^	
AB2	0.34^∗∗∗^	–0.69^∗∗∗^	0.49^∗∗∗^	0.24^∗∗^	–0.33^∗∗^	0.96^∗∗∗^

*NS-not significant; ^∗^*P* < 0.05; ^∗∗^*P* < 0.01; ^∗∗∗^*P* < 0.001; AB1 and AB2 denote AB scores at pod filling and physiological maturity stages, respectively.*

### Identification of Additional SNP Markers within QTLs

**FIGURE 5:**
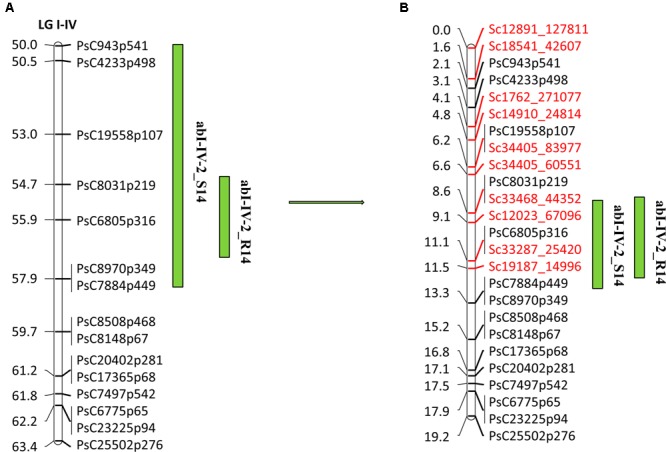
**Quantitative trait locus (QTL) abI-IV-2 based on SNP linkage map in PR-19 (Jha et al., 2016) (A)**, additional SNPs identified by fine mapping using genotyping-by-sequencing (GBS) method in PR-19 **(B)**. Locations of QTLs for AB are shown by vertical bars. S14 and R14 associated with QTLs name denote 2014 Saskatoon and 2014 Rosthern, respectively.

Overall, 10,985 SNPs were identified at a read depth of 10 by selective genotyping of 51 PR-19 RILs using GBS method. After filtering for allele distribution, 6160 SNPs along with 733 previously genotyped SNPs were used for construction of a high density genetic linkage map to identify markers within QTLs. Based on the high density genetic linkage map, 12 SNP markers were identified within abI-IV-2 QTL. Ten of the 12 markers along with previously identified SNP markers from an Illumina GoldenGate array were used for genotyping of a complete set of PR-19 RILs (144) to re-confirm their position and order within the QTL. Following linkage analysis of markers of this QTL, the eight SNP markers identified from the high density genetic linkage map were confirmed to localize within the existing QTL flanked by SNP markers PsC943p541/PsC4233p498 and PsC8970p349/PsC7884p449, whereas two SNPs were located to the region adjacent to the QTL. Mapping of eight additional SNPs within the QTL has increased the map distance of the QTL from 13.4 to 17.1 cM (**Figures 5A,B**).

**FIGURE 6:**
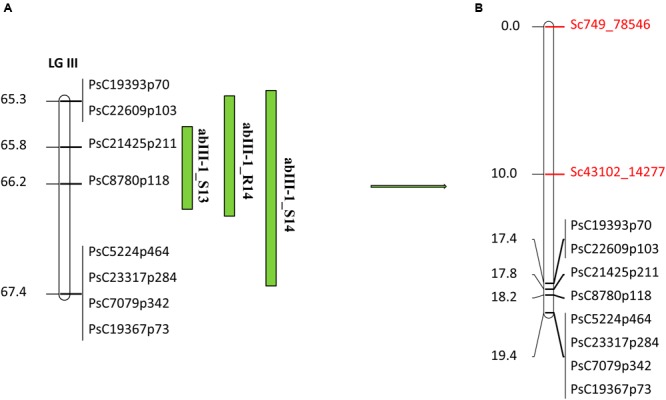
**Quantitative trait locus abIII-1 based on SNP linkage map in PR-19 (Jha et al., 2016) (A)**, additional SNPs identified by fine mapping using GBS method in PR-19 **(B)**. Locations of QTLs for AB are shown by vertical bars. S13, S14, and R14 associated with QTLs name denote 2013 Saskatoon, 2014 Saskatoon, and 2014 Rosthern, respectively.

In the case of abIII-1, based on the high density genetic linkage map, no additional SNP marker was identified within the QTL (**Figure 6B**). Two flanking markers of the QTL were converted to KASP assays and were used for genotyping the complete set of RILs. Linkage analysis of this region based on these two flanking markers and known existing markers within the QTL reconfirmed the order of SNP markers on the high density genetic linkage map.

### Fine Mapping of QTLs for AB Resistance

**FIGURE 7:**
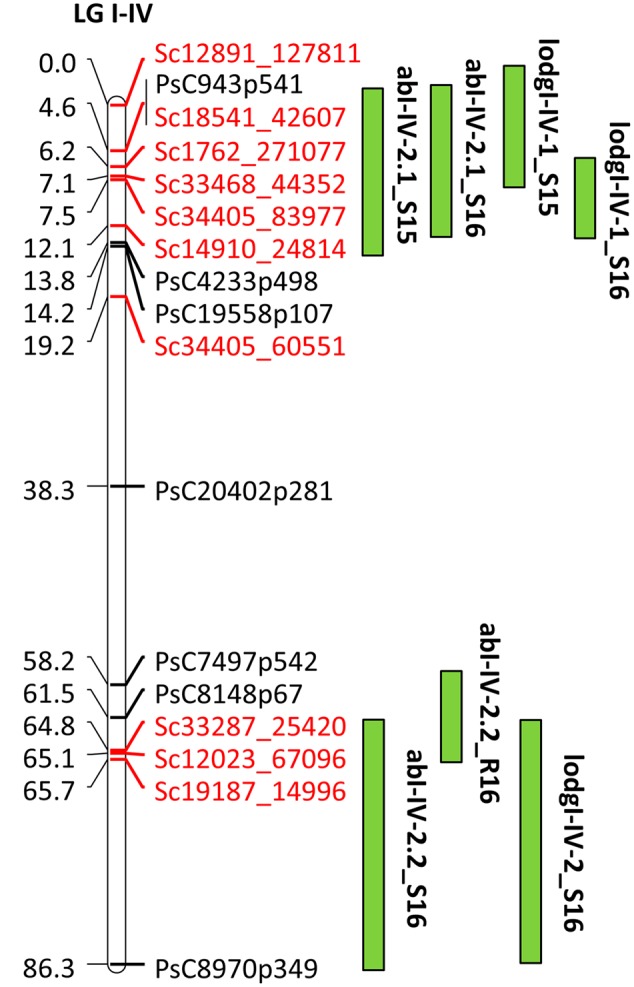
**Quantitative trait locus abI-IV-2 based on GoldenGate and GBS markers in HIF population, HIF-224.** Locations of QTLs for AB and lodging (lodg) are shown by vertical bars. S15, S16, and R16 associated with QTLs name denote 2015 Saskatoon, 2016 Saskatoon, and 2016 Rosthern, respectively.

For fine mapping of abI-IV-2 QTL, 143 lines of HIF-224 segregating for this QTL were genotyped with 20 SNP markers using KASP assays. This set of 20 SNP markers included 10 previously known SNP markers and 10 markers currently identified through the high density genetic linkage map. Of the total genotyped, 17 SNP markers were used for linkage analysis to verify the marker order and distance in the HIF population. The 17 SNP markers represented a map distance of 86.3 cM in HIF-224 population (**Figure 7**). Based on the field evaluation of HIF-224 population in 2015 and 2016 trials, two new QTLs, abI-IV-2.1 and abI-IV-2.2 were identified for AB resistance within the abI-IV-2 QTL (**Table 5** and **Figure 7**). QTL abI-IV-2.1 explained 5.5 to 14% of the total phenotypic variation, whereas abI-IV-2.2 explained 7 to 10% of the total variation. QTLs for lodging resistance were also associated with these two QTLs. Alfetta contributed alleles for AB resistance as well as for lodging resistance. Fine mapping with HIF lines has confirmed the occurrence of AB resistance QTLs within the previously reported QTL ab-IV-2, and provided additional markers for MAS of this QTL in breeding populations.

**Table 5:** Quantitative trait loci (QTLs) detected for reaction to AB resistance and lodging in abI-IV-2 QTL in HIF population, HIF-224 evaluated under field conditions in 2015 at Saskatoon and in 2016 at Saskatoon and Rosthern.

QTL	Trait	Year	Location	Locus^a^	Max. LOD value	% Variation^b^	Additive genetic effect^c^
abI-IV-2.1	AB1, AB2	2015	Saskatoon	Sc1762_271077	6.6	14.0	–0.6
	AB1	2016	Saskatoon	PsC943p541	4.3	5.5	–0.3
lodgI-IV-1	Lodging	2015	Saskatoon	PsC943p541	4.4	9.9	–0.4
	Lodging	2016	Saskatoon	Sc14910_24814	3.7	5.8	–0.3

abI-IV-2.2	AB1, AB2	2016	Saskatoon	PsC8970p349	6.4	9.7	–0.4
	AB1, AB2	2016	Rosthern	Sc33287_25420	5.0	6.6	–0.4
lodgI-IV-2	Lodging	2016	Saskatoon	PsC8970p349	6.8	24.6	–0.1

*AB1 and AB2 denote AB scores at pod filling and physiological maturity stages, respectively. ^a^Closest marker to the identified QTL with maximum LOD value; ^b^Percentage of total variability explained by the QTL detected for the trait; ^c^The value associated with the Alfetta allele; a negative value means that the Alfetta allele decreases the value of the trait.*

Additional SNP makers within the abIII-1 QTL were not identified using the high density genetic linkage map. The extreme distortion of allele segregation determined based on the existing three SNP markers within this QTL did not allow for the determination of the linkage order of these markers in HIF-173 population. Additionally, significant recombination within this QTL was not identified in the HIF family to continue with other tests to determine the significance of these markers.

## Discussion

ABs are the most important diseases of pulse crops (Tivoli et al., 2006; Muehlbauer and Chen, 2007). Resistance breeding is considered the most effective method of control; however, this process is slow due to the complex nature of resistance (Muehlbauer and Chen, 2007; Rubiales and Fondevilla, 2012). Significant progress has been made in resistance breeding with the advancement of innovative tools including next generation sequencing. Several QTLs have been reported for AB resistance in pea (Timmerman-Vaughan et al., 2002, 2004; Tar’an et al., 2003; Prioul et al., 2004; Fondevilla et al., 2008, 2011; Jha et al., 2016), chickpea (Udupa and Baum, 2003; Lichtenzveig et al., 2006; Tar’an et al., 2007; Sabbavarapu et al., 2013), lentil (Sudheesh et al., 2016), and faba bean (Atienza et al., 2016).

Sudheesh et al. (2016) reported validation of previously reported QTLs for AB resistance in lentil on genetic maps based on SNP and SSR markers developed from three RIL populations. Further, they identified two common genomic regions for disease resistance in two out of three maps that could provide validated markers associated with disease for lentil improvement. Similarly, Atienza et al. (2016) studied validation and stability of major QTLs located on chromosomes II and III for AB resistance in faba bean under field and controlled conditions and reported that QTL Af2 located on chromosome II was the same QTL reported previously by other researchers. In chickpea, QTLs were identified for AB resistance on LGs 2, 3, 4, 5, 6, and 8 (Udupa and Baum, 2003; Lichtenzveig et al., 2006; Tar’an et al., 2007; Anbessa et al., 2009; Sabbavarapu et al., 2013). Among them, one major QTL on LG 4 was reported by several researchers under different conditions (Lichtenzveig et al., 2006; Tar’an et al., 2007; Anbessa et al., 2009; Sabbavarapu et al., 2013). Most recently, Li et al. (2017) identified a 100 kb genomic region containing 12 candidate genes for disease resistance associated with a major QTL on chromosome 4 of chickpea using Fst genome-scan and genome-wide association mapping.

Grain yield loss due to AB is a major cause for concern in pea growing regions. Several studies have been conducted to identify improved sources of resistance for pea breeding. Many QTLs were reported for AB resistance in pea (Timmerman-Vaughan et al., 2002, 2004; Tar’an et al., 2003; Prioul et al., 2004; Fondevilla et al., 2008, 2011; Carrillo et al., 2014). Under field conditions, Timmerman-Vaughan et al. (2002, 2004) reported several QTLs for resistance on LGs I, II, III, IV, V, VII, and Group A in two pea mapping populations, whereas Tar’an et al. (2003) identified three QTLs on LGs II, IV, and VI. Prioul et al. (2004) reported six QTLs on LGs III, Va, VI, and VII and 10 QTLs on LGs II, III, Va, and VII under controlled and field conditions, respectively. In *P. sativum* ssp. *syriacum*, six QTLs were reported on LGs II, III, IV and V by Fondevilla et al. (2008), whereas three additional QTLs were identified by Fondevilla et al. (2011) on LGs III and VI. Carrillo et al. (2014) identified four new QTLs on LGs II, III, and V controlling cellular mechanisms involved in AB resistance in *P. sativum ssp. syriacum*. A comparative analysis showed that QTL MpIII.1 (Fondevilla et al., 2008) was located on the same distal part of LG III where Prioul et al. (2004) identified mpIII-1. Fondevilla et al. (2011) indicated that QTLs MpIII.1, MpIII.3, and MpIII.2 detected in *P. sativum* ssp. *syriacum* corresponded to the QTLs mpIII-1, mpIII-3, and mpIII-5 identified in *P. sativum* by Prioul et al. (2004).

With the long-term objective to develop disease resistant pea cultivars, P651 (*P*. *fulvum*) a wild accession with improved resistance was identified and utilized for the development of an inter-specific pea population (PR-19) (Jha et al., 2012; Sindhu et al., 2014). Nine QTLs were identified for AB resistance in PR-19 and these QTLs individually explained 7.5 to 28% of phenotypic variation (Jha et al., 2016). Among these QTLs, abI-IV-2 and abIII-1 were consistent across locations and/or years with greater effects (16 to 28% of the total phenotypic variation) and P651 contributed alleles for disease resistance. Based on shared anchored markers, none of the identified QTLs were located in the regions of previously reported QTLs for AB resistance in pea (Jha et al., 2016).

In this research, abI-IV-2 and abIII-1 were selected for fine mapping to develop closely linked markers associated with AB resistance. For this purpose, four RILs each were identified in the abI-IV-2 and abIII-1 QTLs, for development of HIF populations. Among these RILs, lines PR-19-224 and PR-19-173 were selected for development of HIF-224 and HIF-173, respectively, on the basis of presence of heterozygous alleles as determined by KASP assay and AB scores. These HIFs served as segregating populations for fine mapping.

To find additional markers within QTLs, selective genotyping of 51 PR-19 RILs was performed using GBS. Based on linkage map construction from these RILs, 12 SNPs were identified in regions next to the highly linked markers within QTL abI-IV-2. Ten of the 12 markers were further genotyped on the complete set of PR-19 RILs (144) to determine the exact position and order of the tested markers in the QTL. Eight out of 10 SNPs from GBS were mapped within QTL abI-IV-2. Three markers (Sc34405_60551, Sc33468_44352, and Sc12023_67096) were located within the closest flanking markers (PsC6805p316 and PsC19558p107) located on either side of marker (PsC8031p219) having highest LOD in the QTL. The presence of QTL abI-IV-2 was validated on linkage map of PR-19 lines enriched with additional GBS markers. GBS marker Sc33287_25420 was the closest marker to the identified QTL with maximum LOD value and co-located with PsC6805p316. HIF-224 lines were genotyped using 20 SNPs including 10 GBS markers. A linkage map was constructed from 17 markers which covered 86.3 cM distance. The order and distance of markers were different compared to abI-IV-2 QTL obtained for PR-19. This could be due to recombination between the nearest markers within the QTL, or with markers near this QTL. In the case of PR-19, markers PsC20402p281 and PsC7497p542 were adjacent to the abI-IV-2 QTL and distant (around 7 cM) from the closest marker (PsC8031p219) to the QTL. However, in HIF-224, these markers were present within the QTL abI-IV-2 and covered more than 40 cM distance out of 86.3 cM. The larger map distance in HIF population compared to PR-19 RIL population could be due to the possibility that RIL PR-19-224 selected for HIF development was not heterozygous for the entire QTL. This RIL was fixed for alleles from Alfetta at several loci and was the best line that could be selected for maximum heterozygosity within this QTL based on genotyping of the F_6_ generation. Further, line PR-19-224 selected for HIF development might also contain positive alleles at other ascochyta resistance QTLs which might have affected determining the true effect of this QTL on disease resistance, thus there was no spike observed in LOD value in the HIF population.

Two new QTLs, abI-IV-2.1 and abI-IV-2.2 were identified within abI-IV-2 QTL due to additional SNP markers identified and these QTLs individually explained 5.5 to 14% of the total phenotypic variation. In general, improvement in LOD value was observed in comparison to previously identified QTL. QTLs for lodging resistance were co-located with QTLs associated with AB resistance. The parent Alfetta contributed the alleles for AB resistance as well as for lodging resistance. In this research, it was observed that the difference in AB score was relatively narrow between the parents under field conditions. On a 0-9 scale, Alfetta had 3 to 4 and 4 to 5 disease scores at pod filling and physiological maturity stage, respectively, whereas P651 had 2 to 3 at pod filling stage and 3 to 4 at physiological maturity stage. Further, Alfetta (1–3) had very low lodging score compared to P651 (8–9) on the 1–9 scale. Previous studies reported positive correlation between AB and lodging scores (Tar’an et al., 2003; Banniza et al., 2005; Jha et al., 2013, 2016). Under field conditions, lodging might play an important role in the disease progression and AB score could be related to disease avoidance rather than resistance *per se*. Our previous study (Jha et al., 2016) reported that three out of six QTLs identified under field conditions could account for disease avoidance as these loci were also associated with traits including lodging or plant height. Alternatively, resistance under field conditions could be due to physiological resistance (Khan et al., 2013) as canopy architecture features including branching, lodging resistance, and leaf area index could affect the impact of disease by affecting the microclimate within the canopy and splash dispersal of *P. pinodes* conidia (Schoeny et al., 2008; Le May et al., 2009). In case of abIII-1, no additional marker was identified within the QTL (2.1 cM). Five and three additional SNP markers identified in QTLs abI-IV-2.1 and abI-IV-2.2, respectively, by fine mapping can be used for marker assisted selection. Further, promising HIF lines harboring QTLs for disease resistance can be utilized as donors for development of cultivars with improved AB resistance.

## Author Contributions

AJ, BT, and TW conceived and designed the experiments. AJ and TW were involved in the development of HIFs and the multi-year field trials. GBS markers were developed by KG. AJ and KG were involved in the genotyping of PR-19 and HIFs and data analysis. AJ wrote the manuscript with input from KG, TW, and BT. All authors have read and approved the final manuscript.

## Conflict of Interest Statement

The authors declare that the research was conducted in the absence of any commercial or financial relationships that could be construed as a potential conflict of interest.
